# Obituary

**Published:** 1876-11

**Authors:** 


### Obituary.

JULIAN S. SHERMAN, A. M., M. D., Prof. of Orthopedic Surgery and Diseases of the Joints, in the Chicago Medical College, died on the 16th of August, 1876.

Prof. Sherman was born in Quincy, Ill. At the age of fourteen he entered the Russell Military Institute at New Haven, Conn., where he remained two years. He then entered St. Paul's College, Mo., where, in due time, he received the two degrees of Bachelor of Arts and of Master of Arts. He commenced the study of medicine with Drs. Johnson and Andrews, of Chicago, and graduated in the Chicago Medical College in 1864. He entered upon practice in the same city, and soon developed such a talent as a lecturer, and so much capacity as an orthopedic surgeon, that he was made Professor of his favorite branch.

Prof. Sherman made numerous improvements in his department. Among his inventions were a superior spinal supporter for Pott's Disease, and an improved brace for disease of the knee, doing away with some of the serious objections which lie against the instruments made in New York. Soon after he commenced his career as a successful lecturer in the College, he had the misfortune to be attacked with an abdominal abscess, which, in a great measure disabled him for duty for two years. Before he recovered from this he met with an accident by the explosion of some chemicals in a bottle which destroyed one eye, and, in connection with the previous trouble, seriously impaired his health. His death has deprived his colleagues of a genial and noble-hearted co-laborer, and the College of an able and attractive lecturer, and has carried sorrow to a large circle of true friends. E. A.

